# Supplementary figures and images for: Hepatic inactivation of murine Surf4 results in marked reduction in plasma cholesterol
Source: eLife. 2022 Oct 4;11:e82269. doi: 10.7554/eLife.82269 (PMC9581532; doi:10.7554/eLife.82269)

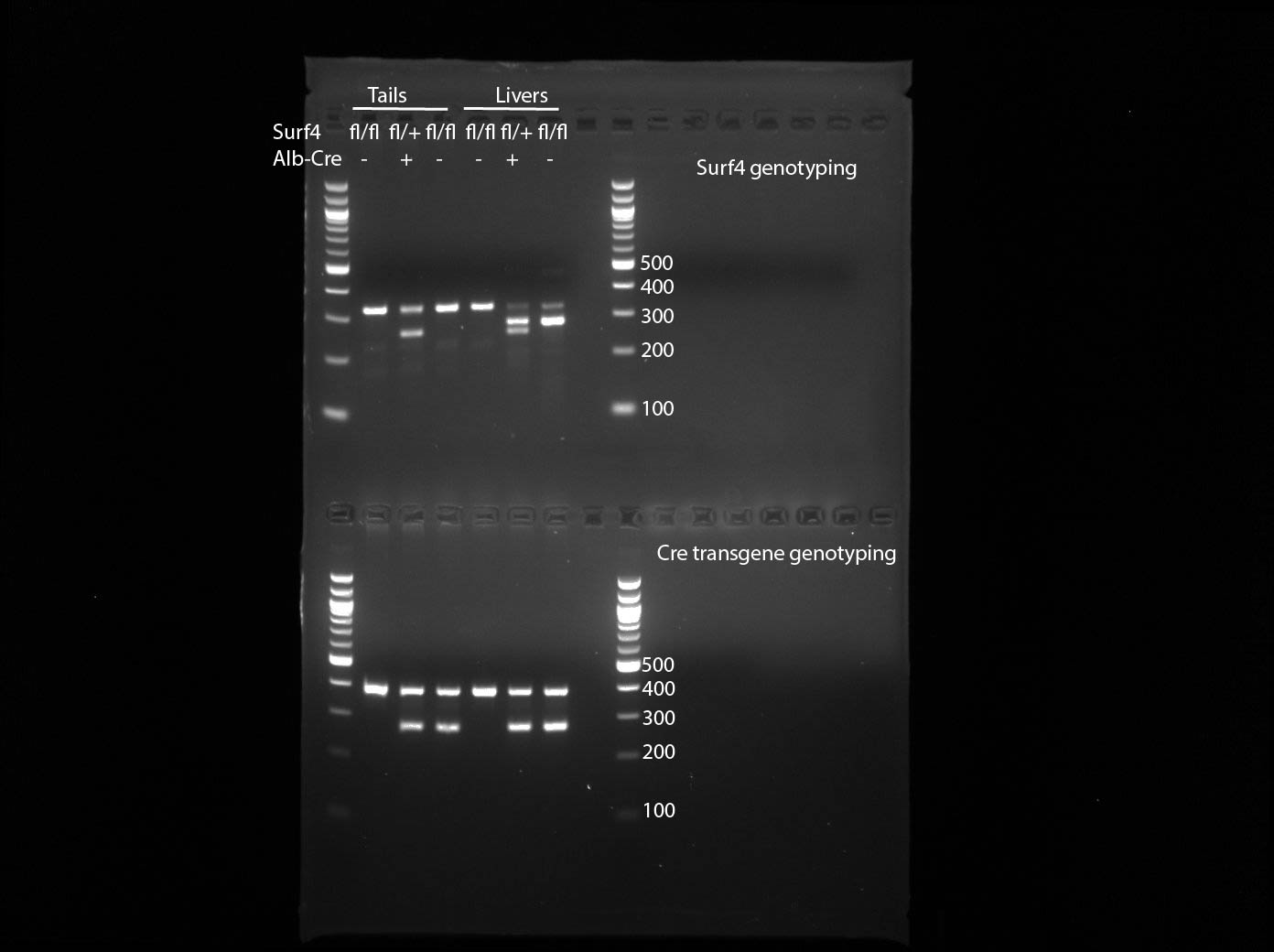

Supplement: Figure 1—source data 1. [file elife-82269-fig1-data1.zip › figure1-source-data/fig1b-genotyping_gel_annotated.jpg]

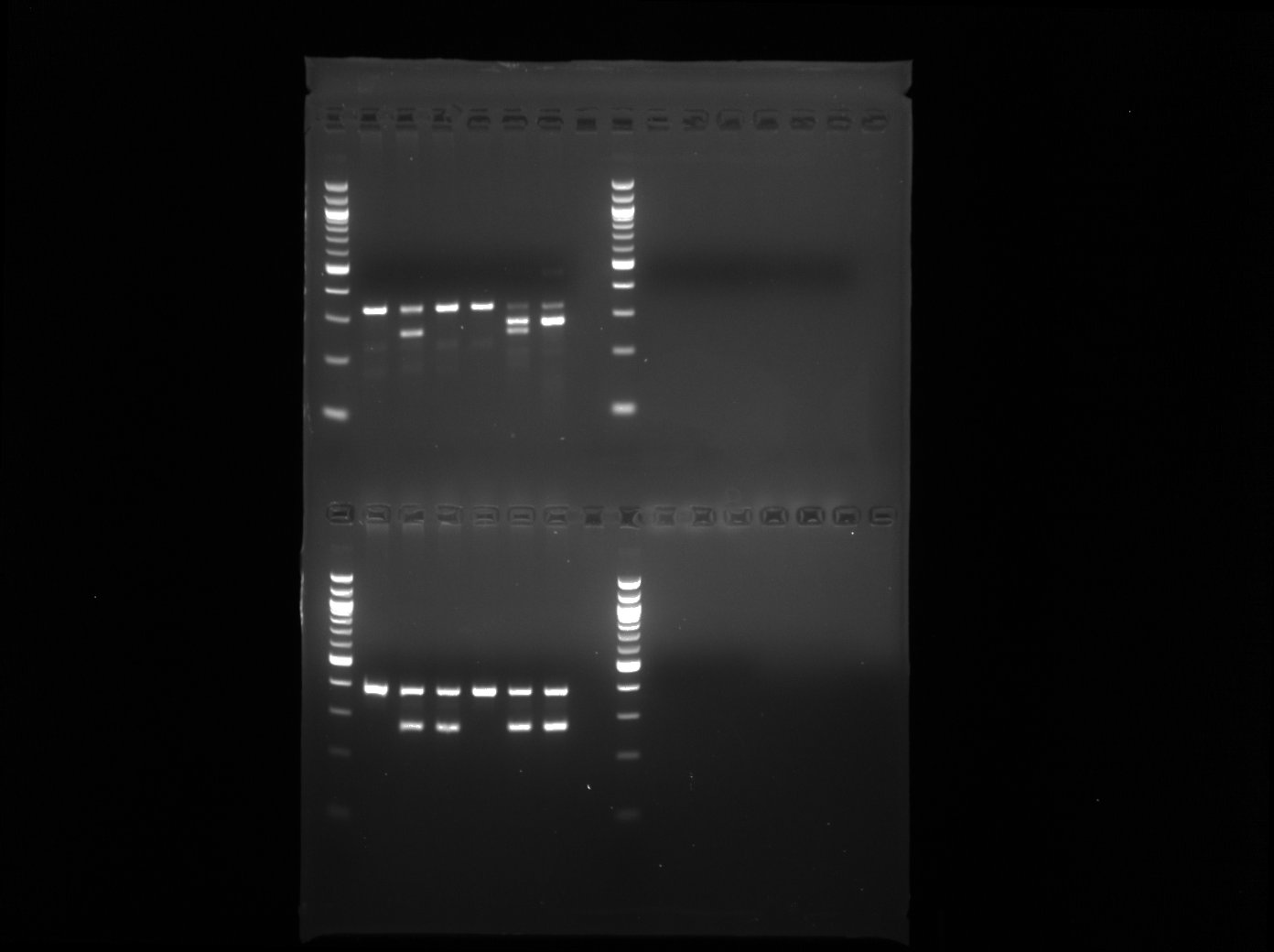

Supplement: Figure 1—source data 1. [file elife-82269-fig1-data1.zip › figure1-source-data/fig1b-genotyping_gel_unedited.jpg]

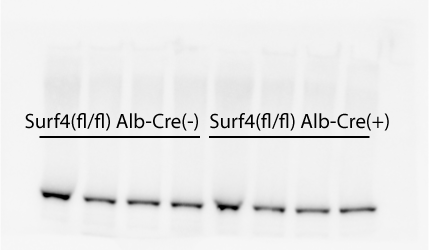

Supplement: Figure 2—source data 1. [file elife-82269-fig2-data1.zip › figure2-source-data/fig2b-liver_hsp90_annotated.tif]

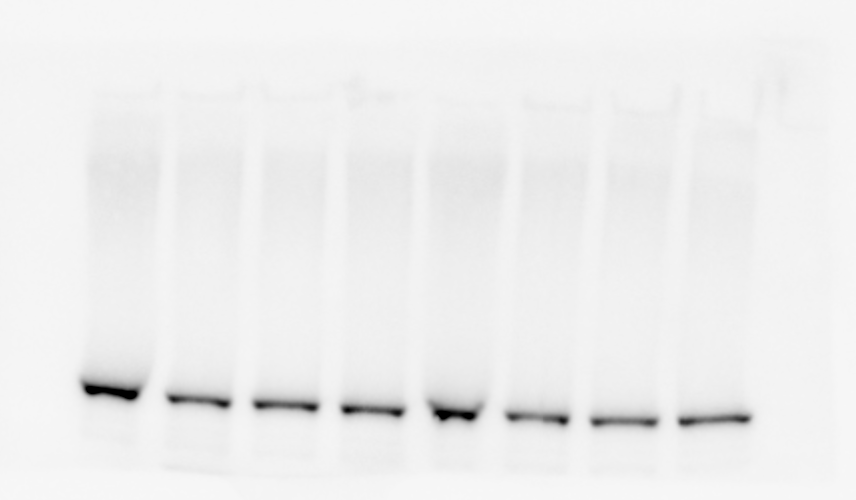

Supplement: Figure 2—source data 1. [file elife-82269-fig2-data1.zip › figure2-source-data/fig2b-liver_hsp90_unedited.tif]

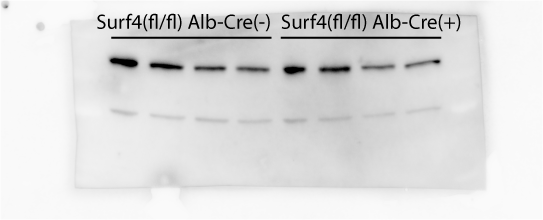

Supplement: Figure 2—source data 1. [file elife-82269-fig2-data1.zip › figure2-source-data/fig2b-liver_pcsk9_annotated.tif]

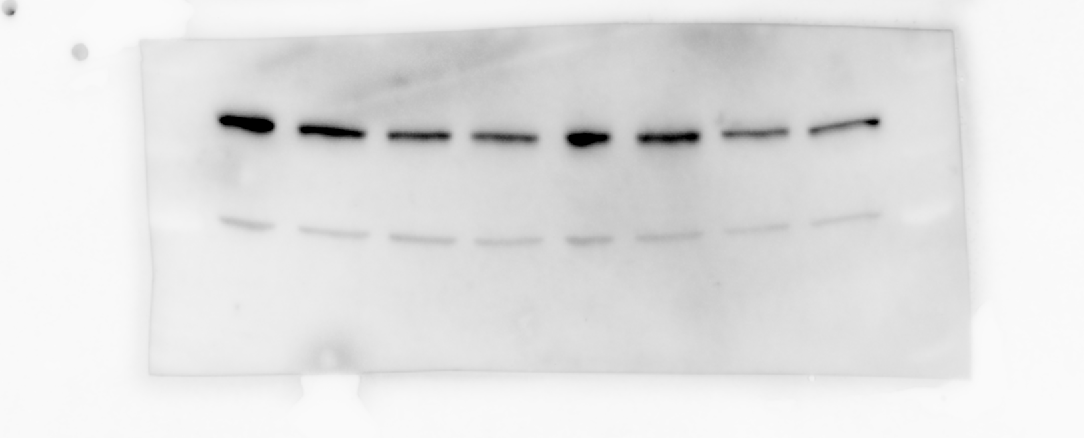

Supplement: Figure 2—source data 1. [file elife-82269-fig2-data1.zip › figure2-source-data/fig2b-liver_pcsk9_unedited.tif]

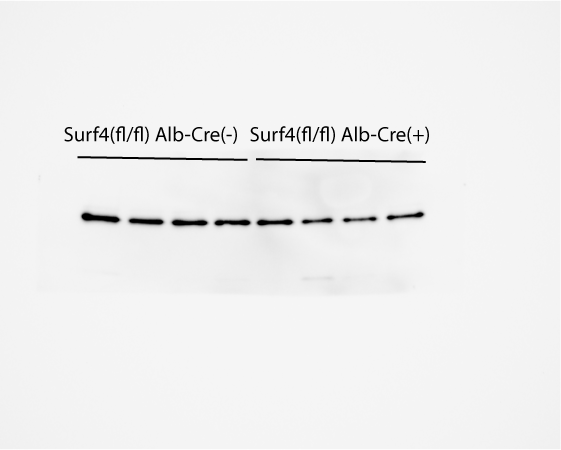

Supplement: Figure 2—source data 1. [file elife-82269-fig2-data1.zip › figure2-source-data/fig2e-liver_gapdh_annotated.tif]

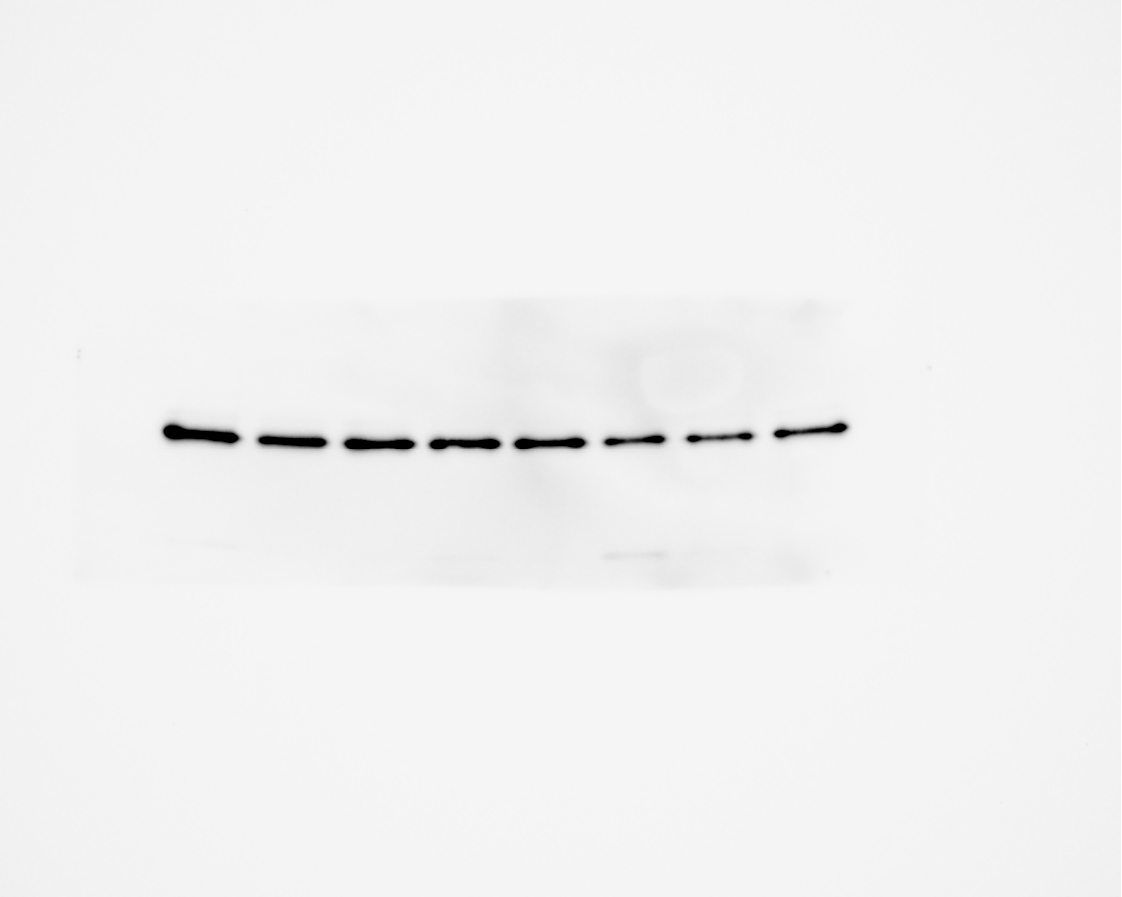

Supplement: Figure 2—source data 1. [file elife-82269-fig2-data1.zip › figure2-source-data/fig2e-liver_gapdh_unedited.tif]

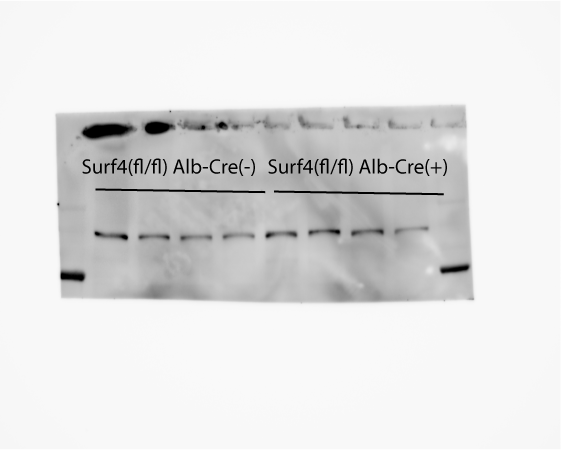

Supplement: Figure 2—source data 1. [file elife-82269-fig2-data1.zip › figure2-source-data/fig2e-liver_ldlr_annotated.tif]

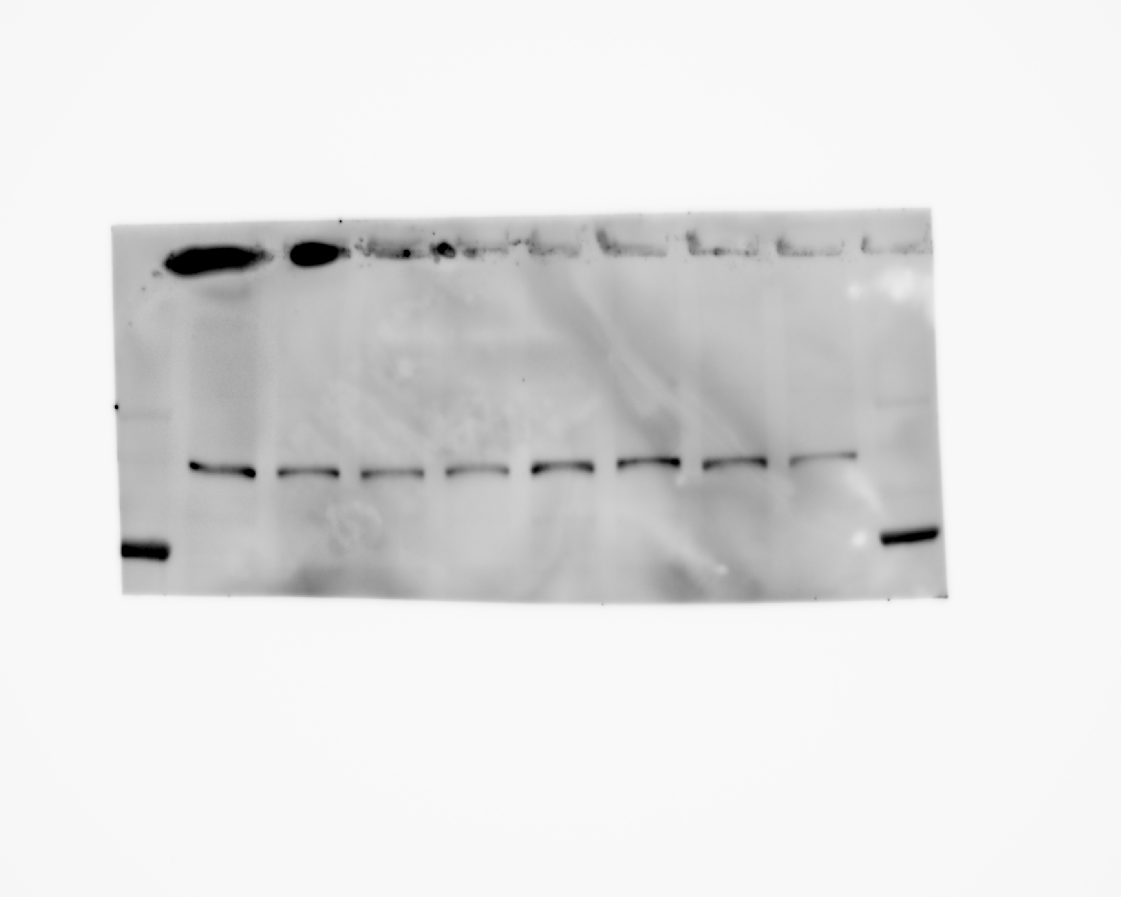

Supplement: Figure 2—source data 1. [file elife-82269-fig2-data1.zip › figure2-source-data/fig2e-liver_ldlr_unedited.tif]

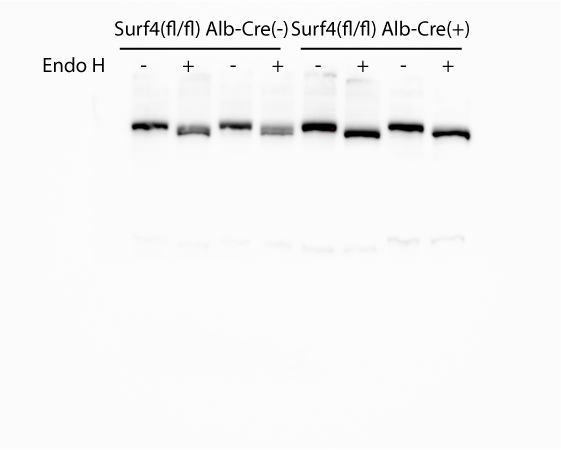

Supplement: Figure 3—source data 1. [file elife-82269-fig3-data1.zip › figure3-source-data/fig3b-source-data_apob_gel1_annotated.tif]

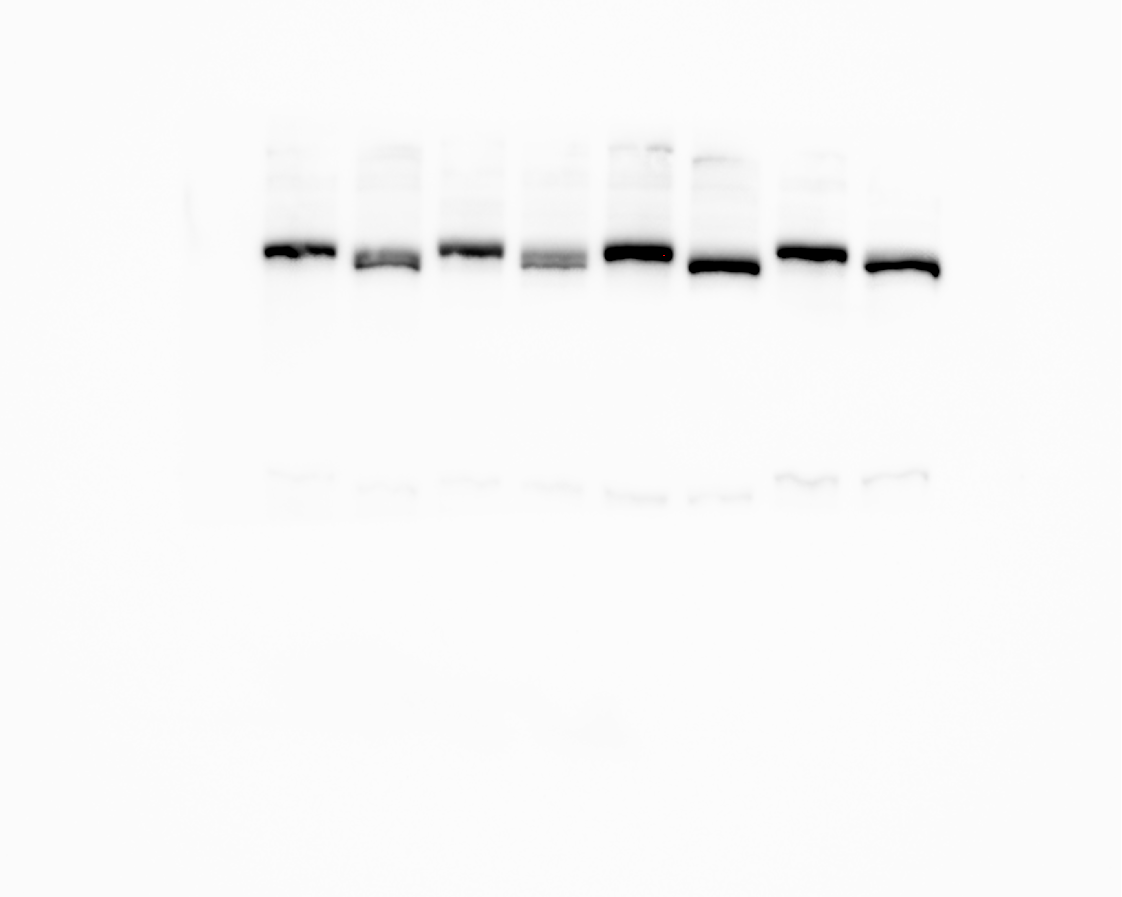

Supplement: Figure 3—source data 1. [file elife-82269-fig3-data1.zip › figure3-source-data/fig3b-source-data_apob_gel1_unedited.tif]

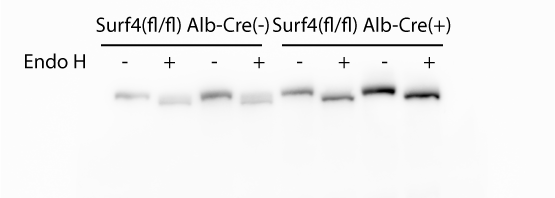

Supplement: Figure 3—source data 1. [file elife-82269-fig3-data1.zip › figure3-source-data/fig3b-source-data_apob_gel2_annotated.tif]

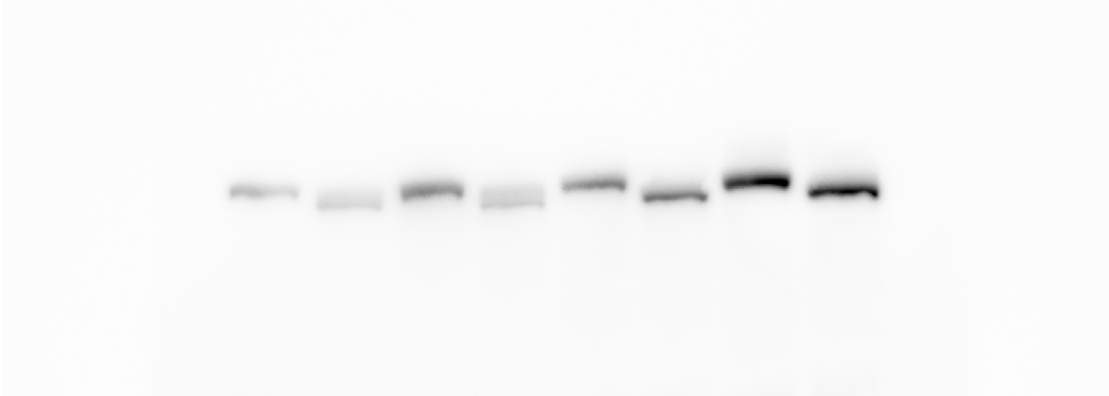

Supplement: Figure 3—source data 1. [file elife-82269-fig3-data1.zip › figure3-source-data/fig3b-source-data_apob_gel2_unedited.tif]

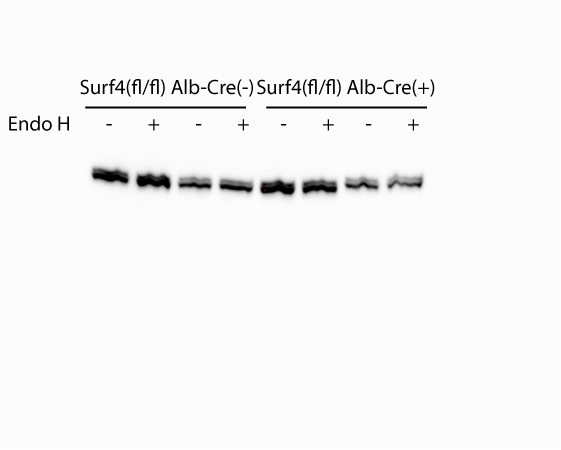

Supplement: Figure 3—source data 1. [file elife-82269-fig3-data1.zip › figure3-source-data/fig3b-source-data_hsp90_gel1_annotated.tif]

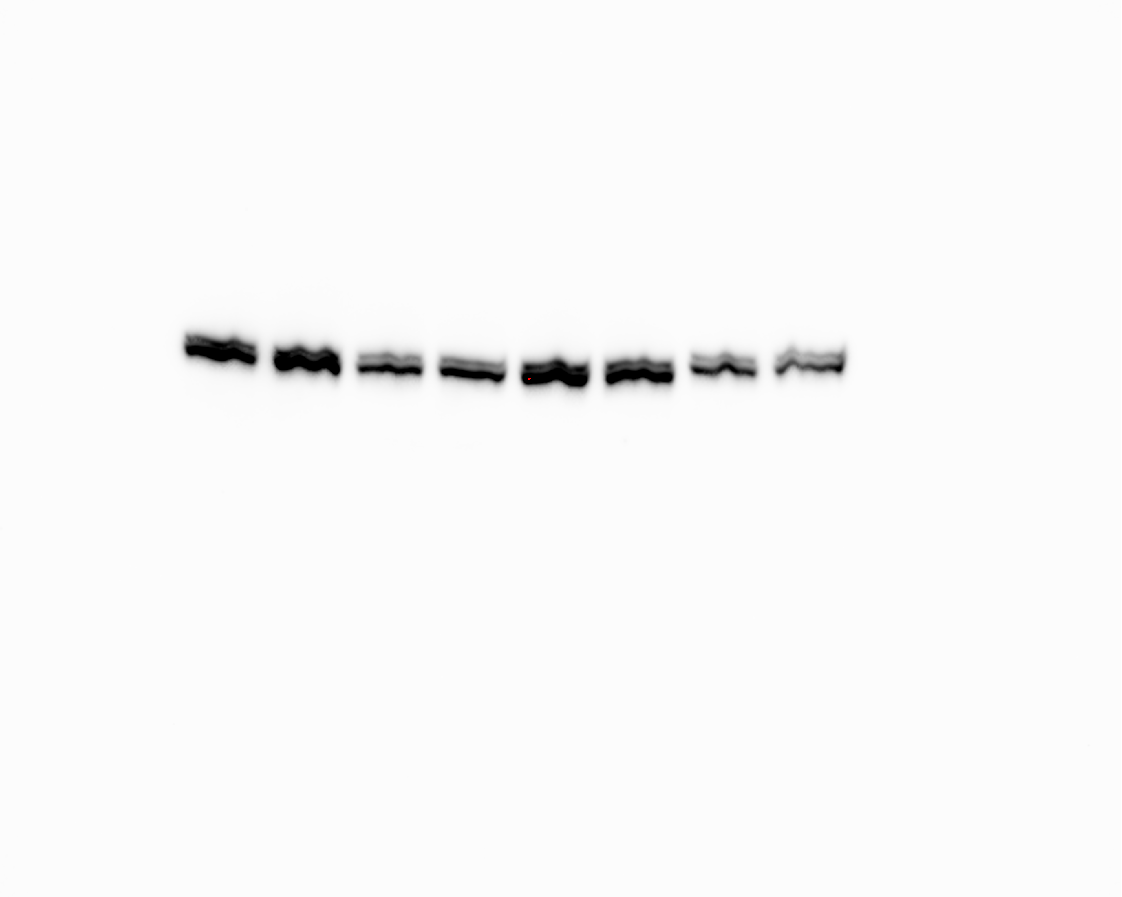

Supplement: Figure 3—source data 1. [file elife-82269-fig3-data1.zip › figure3-source-data/fig3b-source-data_hsp90_gel1_unedited.tif]

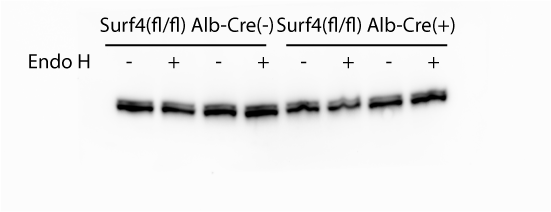

Supplement: Figure 3—source data 1. [file elife-82269-fig3-data1.zip › figure3-source-data/fig3b-source-data_hsp90_gel2_annotated.tif]

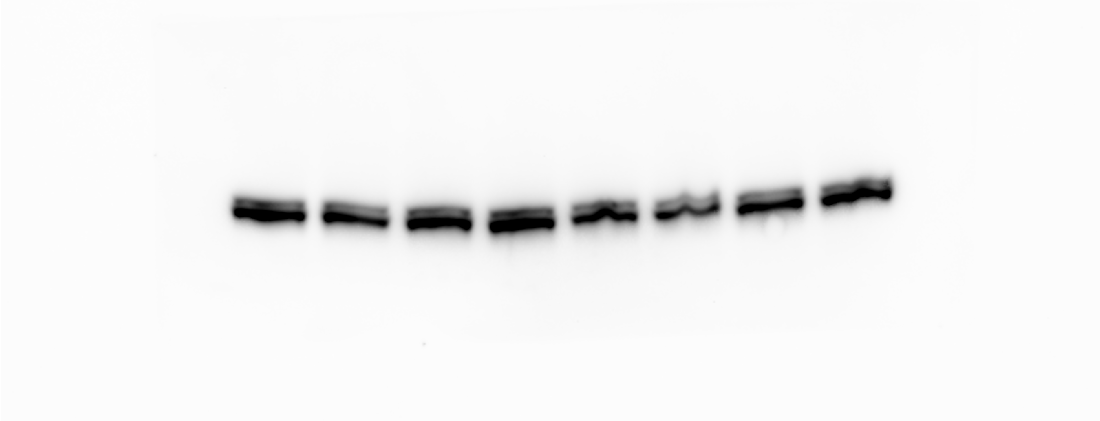

Supplement: Figure 3—source data 1. [file elife-82269-fig3-data1.zip › figure3-source-data/fig3b-source-data_hsp90_gel2_unedited.tif]

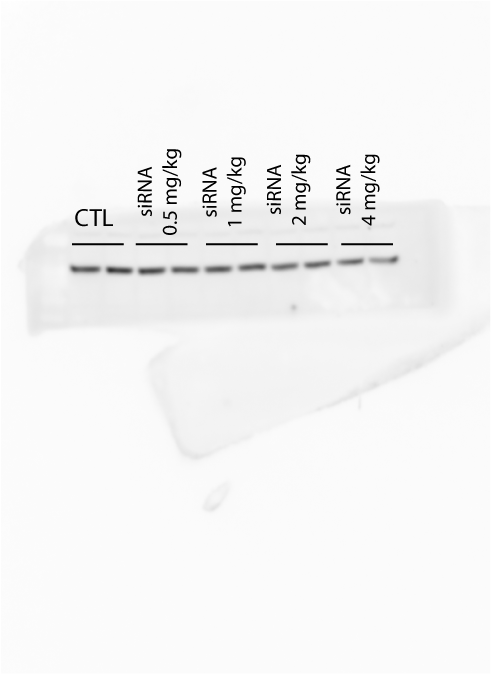

Supplement: Figure 6—figure supplement 1—source data 1. [file elife-82269-fig6-figsupp1-data1.zip › figures5-source-data/figures5-albumin_annotated.tif]

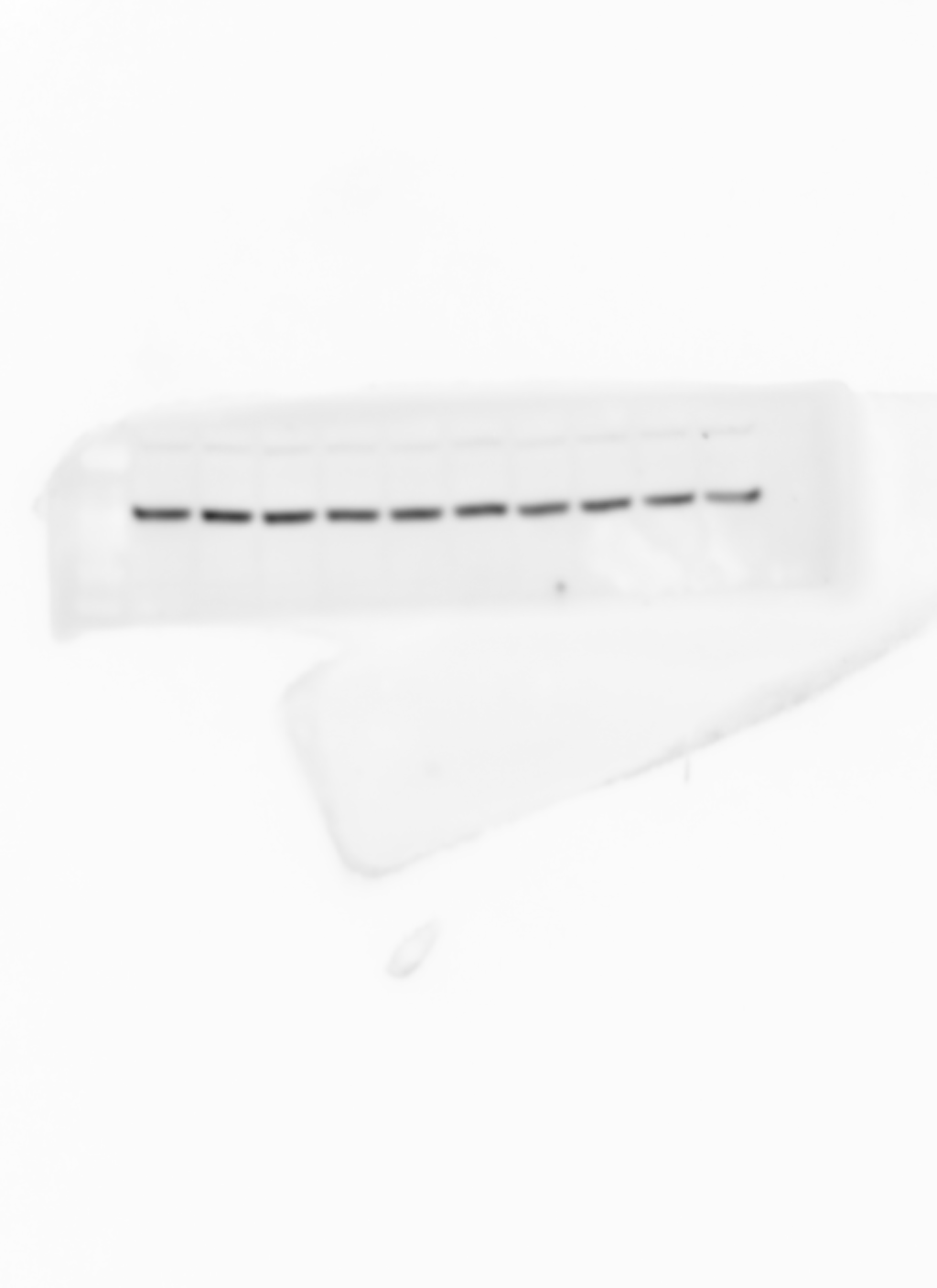

Supplement: Figure 6—figure supplement 1—source data 1. [file elife-82269-fig6-figsupp1-data1.zip › figures5-source-data/figures5-albumin_unedited.tif]

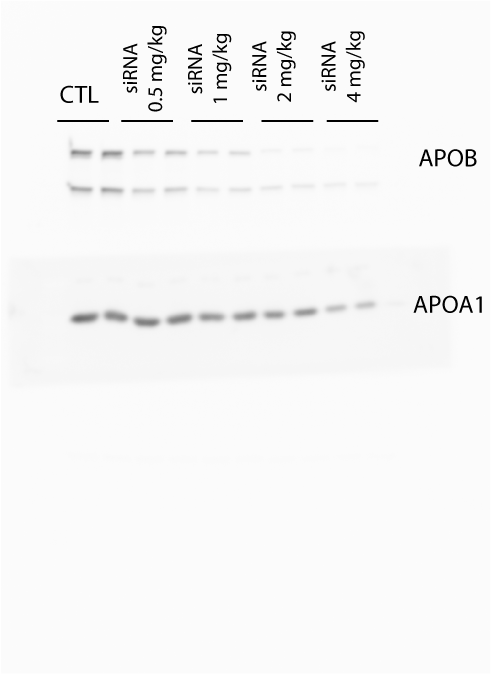

Supplement: Figure 6—figure supplement 1—source data 1. [file elife-82269-fig6-figsupp1-data1.zip › figures5-source-data/figures5-APOB(up)-APOA1(down)_annotated.tif]

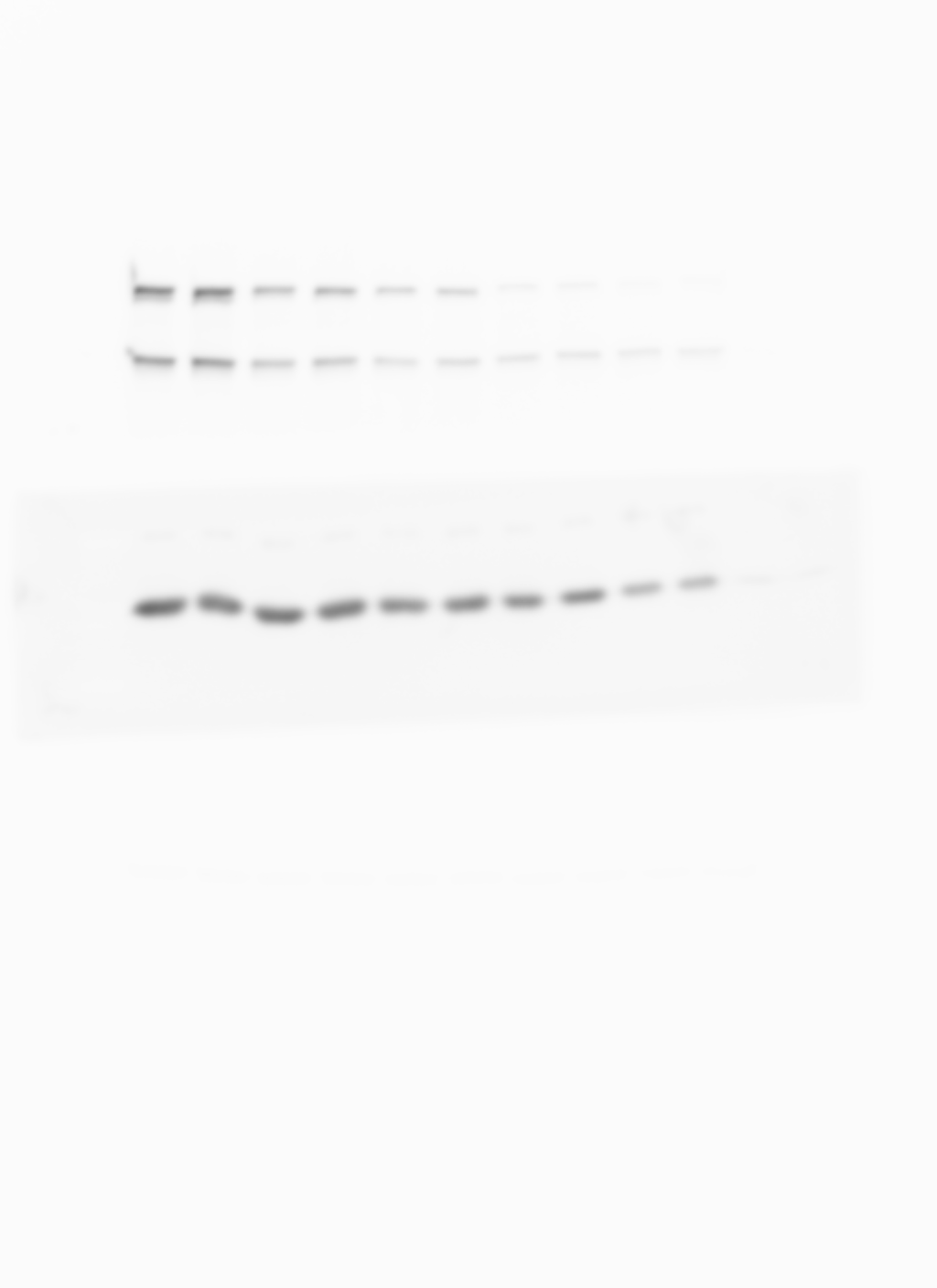

Supplement: Figure 6—figure supplement 1—source data 1. [file elife-82269-fig6-figsupp1-data1.zip › figures5-source-data/figures5-APOB(up)-APOA1(down)_unedited.tif]

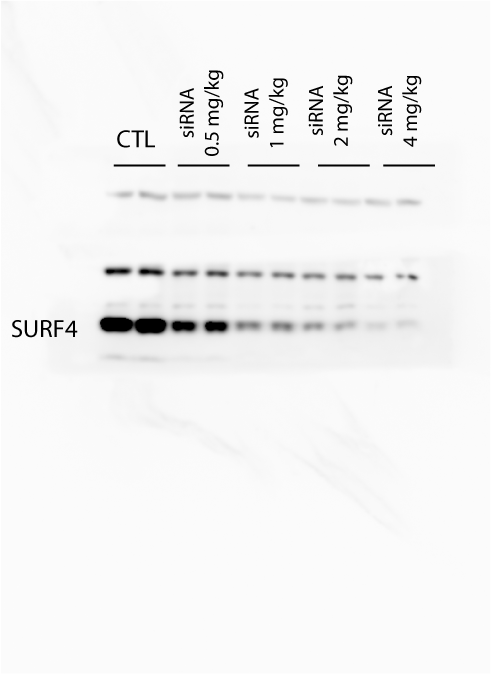

Supplement: Figure 6—figure supplement 1—source data 1. [file elife-82269-fig6-figsupp1-data1.zip › figures5-source-data/figures5-SURF4(down)_annotated.tif]

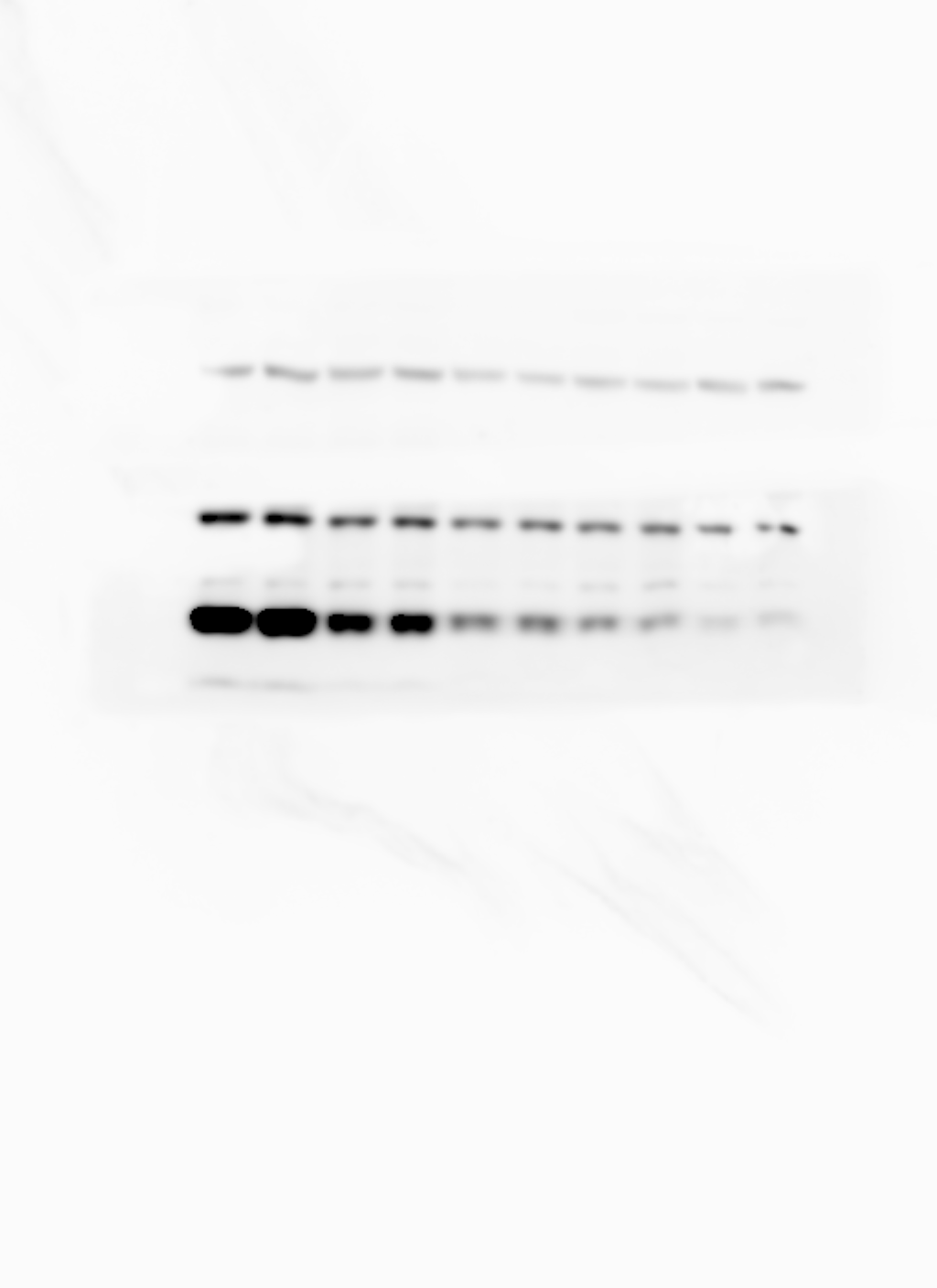

Supplement: Figure 6—figure supplement 1—source data 1. [file elife-82269-fig6-figsupp1-data1.zip › figures5-source-data/figures5-SURF4(down)_unedited.tif]

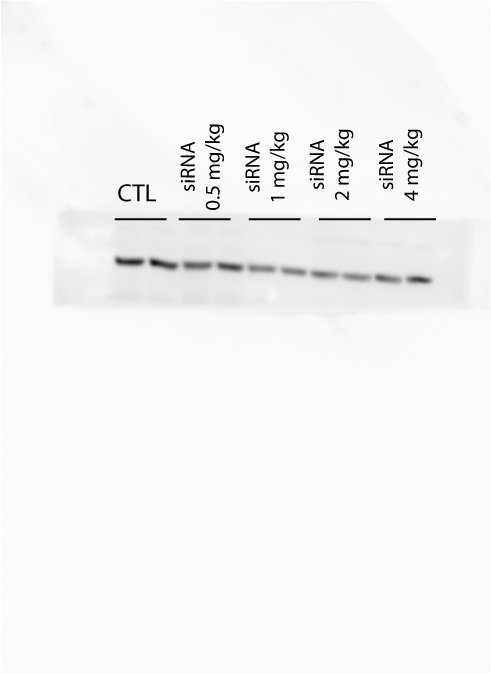

Supplement: Figure 6—figure supplement 1—source data 1. [file elife-82269-fig6-figsupp1-data1.zip › figures5-source-data/figures5-Tubulin_annotated.tif]

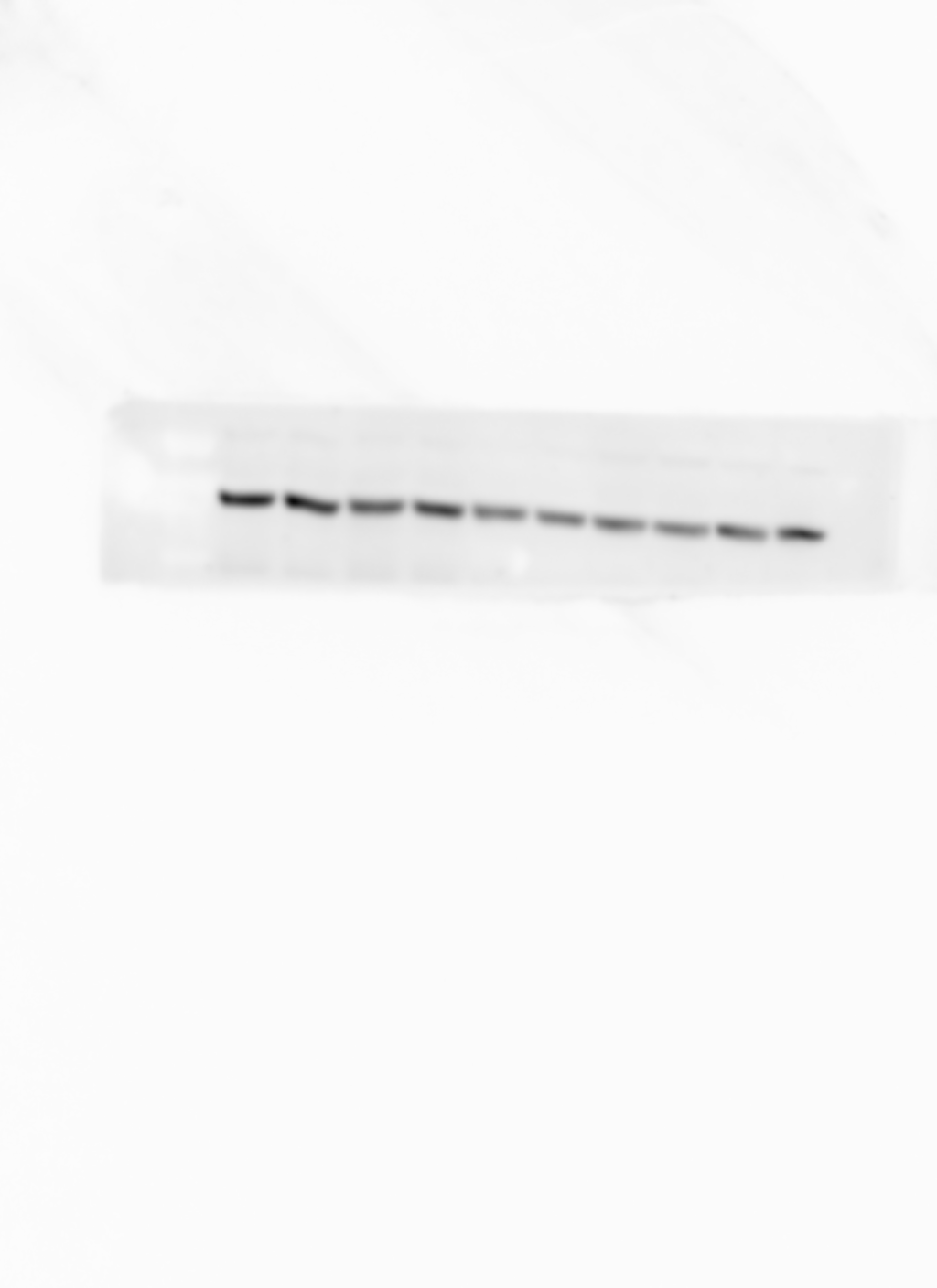

Supplement: Figure 6—figure supplement 1—source data 1. [file elife-82269-fig6-figsupp1-data1.zip › figures5-source-data/figures5-Tubulin_unedited.tif]
