# Supplementary material for: Hepatic inactivation of murine Surf4 results in marked reduction in plasma cholesterol
Source: eLife. 2022 Oct 4;11:e82269. doi: 10.7554/eLife.82269 (PMC9581532; doi:10.7554/eLife.82269)
Supplement: Supplementary file 1. [file elife-82269-supp1.docx]

**Supplement File 1. Primers used in this study**

| **Primer name** | **Target** | **Sequence (5’ 🡪 3’)** |
| --- | --- | --- |
| Surf4-fl-F1 (P1) | *Surf4* | TGAGAAAAGTATGGAGTCCACCTGC |
| Surf4-fl-R1 (P2) | *Surf4* | AACAGCTGTGCTATGGGCTGGAAAG |
| Surf4-fl-R2 (P3) | *Surf4* | CCTGCCTCTAAATCCCAAATGCTGTCG |
| Cre-genotyping-F1 | *Cre transgene* | CCATCTGCCACCAGCCAG |
| Cre-genotyping-R1 | *Cre transgene* | TCGCCATCTTCCAGCAGG |
| Cre-genotyping-F2 | *Cpxm1* (Control) | ACTGGGATCTTCGAACTCTTTGGAC |
| Cre-genotyping-R2 | *Cpxm1* (Control) | GATGTTGGGGCACTGCTCATTCACC |
| Alb-cre-F1 | *Alb-Cre* | TGCAAACATCACATGCACAC |
| Alb-cre-R1 | *Alb-Cre* | TTGGCCCCTTACCATAACTG |
| Alb-cre-R2 | *Alb-Cre* | GAAGCAGAAGCTTAGGAAGATGG |
| Surf4-qPCR-F1 | *Surf4* (exon 2) | CTGTTGGCCTCATCCTTCGT |
| Surf4-qPCR-R1 | *Surf4* (exon 3) | GGCAATTGTCTGCAGTGCG |
| Surf4-qPCR-F2 | *Surf4* (exon 5) | TTTGCTGGTGTCCCAACCAT |
| Surf4-qPCR-R2 | *Surf4* (exon 6) | AGCTGTGCCCACAATGTTCT |
| Pcsk9-qPCR-F1 | *Pcsk9* | TATAGCCGCATCCTCAACGC |
| Pcsk9-qPCR-R1 | *Pcsk9* | CCCGACTGTGATGACCTCTG |
| Rpl37-F | *Rpl37* | CGGGACTGGTCGGATGAG |
| Rpl37-R | *Rpl37* | TCACGGAATCCATGTCTGAATC |
| Gapdh-F | *Gapdh* | ACCCAGAAGACTGTGGATGG |
| Gapdh-R | *Gapdh* | ACACATTGGGGGTAGGAACA |
