# Supplementary material for: Hepatic inactivation of murine Surf4 results in marked reduction in plasma cholesterol
Source: eLife. 2022 Oct 4;11:e82269. doi: 10.7554/eLife.82269 (PMC9581532; doi:10.7554/eLife.82269)
Supplement: Supplementary file 2. [file elife-82269-supp2.docx]

**Supplement File 2. Guide RNA sequences for CRISPR-mediated *in vivo* hepatic *Surf4* inactivation**

|  | **Guide RNA sequence (5’-3’)** |
| --- | --- |
| Control | TGCGAATACGCCCACGCGAT |
| *Surf4g1* | ACAGAACGACCTGATGGGCA |
| *Surf4g2* | GCATCCGCATGTGGTTCCAG |
| *Surf4g3* | CAGCAGGTTGAGGAACACGA |
