# Supplementary material for: Hepatic inactivation of murine Surf4 results in marked reduction in plasma cholesterol
Source: eLife. 2022 Oct 4;11:e82269. doi: 10.7554/eLife.82269 (PMC9581532; doi:10.7554/eLife.82269)
Supplement: Supplementary file 3. [file elife-82269-supp3.docx]

**Supplement File 3. Sequences and modifications of oligonucleotides for siRNA in mice**

|  | **Sense (5’-3’)**  **3' GalNAc conjugated** | **Antiense (5’-3’)**  **3' GalNAc conjugated** |
| --- | --- | --- |
| si*Surf4*-1 | CmsCmsAmUmCfAmAfCfGfUmGmUmAmUmUmUmCmAmAm | UmsUfsGmAmAmAfUmAfCfAmCmGmUmUfGmAfUmGmGmsCmsAm |
| si*Surf4*-2 | GmsGmsAmCmAfAmUfCfCfCmGmGmUmAmUmAmUmAmAm | UmsUfsAmUmAmUfAmCfCfGmGmGmAmUfUmGfUmCmCmsAmsGm |
| siCTL | mU*mU*mCmUfCmCfGfAfAmC  mGmUmGmUmCmAmCmGmU | mA*fC*mGmUmGfAmCfAfCmGmUmUmCfGmGfAmGmAmA*mU*mU |
